# Temporal Patterns of Eating and Diet Composition of Night Shift Workers Are Influenced More by Shift Type than by Chronotype

**DOI:** 10.3390/nu17223561

**Published:** 2025-11-14

**Authors:** Yan Yin Phoi, Jillian Dorrian, Michelle Rogers, Gloria K. W. Leung, Rochelle Davis, Angela B. Clark, Corinne Davis, Maxine P. Bonham, Alison M. Coates

**Affiliations:** 1 Allied Health and Human Performance, Alliance for Research in Exercise, Nutrition and Activity (ARENA) Research Centre, University of South Australia, Adelaide, SA 5001, Australia; yan_yin.phoi@mymail.unisa.edu.au; 2 Justice and Society, Behaviour-Brain-Body Research Centre, University of South Australia, Adelaide, SA 5072, Australia; jill.dorrian@unisa.edu.au (J.D.); michelle.rogers@sahmri.com (M.R.); 3 Department of Nutrition, Dietetics & Food, Monash University, Melbourne, VIC 3168, Australia; g.leung@deakin.edu.au (G.K.W.L.); rochelle.davis@monash.edu (R.D.); abclark@swin.edu.au (A.B.C.); corinne.davis@monash.edu (C.D.); maxine.bonham@monash.edu (M.P.B.)

**Keywords:** chrononutrition, temporal patterns of eating, chronotype, shift workers, nutrient intake, diet composition

## Abstract

**Background/Objectives:** Shift work and chronotype influence timing and type of food consumed, yet their combined influence is unclear. This study determined differences between temporal patterns of eating (times of first (FEO), last (LEO), and largest eating occasions (LarEO), duration of eating window (DEW), eating frequency) and nutrient intake of night shift workers on and off shift and the additional influence of chronotype. **Methods:** Participants (46.6 ± 10.2 years, BMI: 33.9 ± 5.6 kg/m^2^, male/female: 57/72) completed work/sleep/food diaries, and the Composite Scale of Morningness. Dietary profiles were characterized by day type as follows: morning shift (MS), 1st night shift (1stNS), subsequent night shifts (SNS), 1st day off after night shifts (1stDONS), or other days off (DO). **Results:** Across day types, there were significant differences in FEO (*p* < 0.001), LEO (*p* < 0.001), LarEO (*p* = 0.025), DEW (*p* < 0.001), eating frequency (*p* = 0.003), total energy (*p* = 0.022), and fibre intake (*p* < 0.001). Compared to MS, all night shifts had later FEO, LEO, and LarEO; 1stNS had longer DEW and higher fibre but no differences in frequency, energy, and macronutrient intake. Greater morningness was associated with earlier FEO, LEO, LarEO, and lower %energy from fat and saturated fat. Effect of chronotype on temporal eating patterns was not different across day types; there was a significant, positive relationship between morningness and %energy from carbohydrate (%Energy_CHO_) on MS (*p* = 0.004) and 1stDONS (*p* = 0.040). **Conclusions:** Dietary habits of night shift workers vary by shift schedule and degree of morningness. Further studies will confirm if shift schedule is more influential than chronotype on shift workers’ dietary habits.

## 1. Introduction

Our circadian system is governed by a central clock in the suprachiasmatic nucleus in synchrony with peripheral clocks located throughout body in response to the 24 h cycle of light and dark [1]. Timing and composition of food intake serve as cues to peripheral clocks, and can affect the regulation of metabolic processes [1]. Shift workers who work night shifts have increased frequency of eating at night, consume a greater amount of energy later in the day, and report a greater overall intake of sugar and alcohol than those who work morning shifts [2,3,4]. Eating during the night conflicts with the diurnal regulation of our circadian system, and reflects as impairments in glucose tolerance [5] and lipid metabolism [6], which contributes to the elevated risk of developing obesity, diabetes, and cardiovascular disease observed amongst night shift workers [7,8,9].

Chrononutrition is the study of timing, frequency, and regularity of eating behaviour [10] on health [11]. A previous scoping review [12] and Delphi study [13] involving experts in circadian biology, agreed that in research focusing on chrononutrition in any population, outcomes should include times of first (FEO), last (LEO), and largest eating occasion (LarEO); duration of eating window (DEW); and frequency of eating (meal, snack, and total frequency). For shift-working populations, examining differences in patterns between shift types and days off was also highly relevant [13]. However, while previous studies have described some of these temporal aspects of eating across each shift type, sample sizes have been small, limiting generalizability of findings [2,4]. Confirming if there are differences in food consumption habits within the wake window (time period between wake time and sleep time) of shift workers between shift types and days off will allow for shift-specific dietary advice and workplace support to be provided by healthcare professionals and employers.

Additionally, intrinsic to each individual is their chronotype, which describes the behavioural manifestation of their circadian rhythm, including times that activities such as sleeping and eating occur when unbound by societal pressures such as start and end times of work [14]. Previous reviews have consistently shown that morning chronotypes tend to have earlier meals while evening chronotypes tend to skip breakfast and distribute a greater amount of food intake towards later parts of the day [12,15]. Furthermore, chronotype appears to influence food choice, with evening chronotypes making less healthy dietary choices, consisting of more sugar-sweetened products, caffeine, and alcohol, and less vegetables than morning chronotypes [15,16]. Given the impact of timing and type of food intake on nutrient metabolism, it has been postulated that the greater incidence of overweight and obesity, risk of diabetes, and hypertension observed amongst evening chronotypes compared to morning chronotypes can be at least in part attributed to their dietary behaviour [16,17].

Of interest are previous observations that different chronotypes adapt their lifestyle behaviours differently when exposed to shift work. Evening chronotypes present with shorter sleep duration, poorer sleep quality, and greater need for recovery than morning chronotypes on morning shifts, with the reverse observed for morning chronotypes when performing night shifts [18,19]. Furthermore, when individuals’ circadian preference is mismatched with shift type (e.g., morning chronotypes working night shifts or evening chronotypes working day shifts), they face a 1.3-fold greater risk of prostate cancer, 3.9 times greater odds of developing breast cancer, and 1.5 times greater odds of developing diabetes [20,21,22]. On the contrary, short-term studies that deliberately matched chronotype with shift schedule (e.g., morning chronotypes work morning shifts) observed that participants experienced better sleep duration and sleep quality for both chronotypes [23,24]. Similarly, it is possible that a mismatch between circadian preference (chronotype) and shift type may also affect temporal eating patterns and nutrient intake, which may contribute to worsened cardiometabolic health outcomes. However, few studies have examined whether and how chronotype influences dietary patterns across shifts. A simulated shift work study found no differences between chronotypes in hunger, desire to eat fruit or fast food, or actual snack consumption before and during a night shift [25]. Other studies of shift workers investigated associations between chronotype and food groups [26], chronotype and temporal patterns of eating/meal content [27], or energy and nutrient intakes of individuals based on whether shift type was matched or mismatched with chronotype [28]. In all of these studies, food consumption patterns, including specific temporal aspects of eating, were not characterized by shift type, and the influence of chronotype across each shift type was not described.

Hence, this study aims to (1) characterize times of FEO, LEO, LarEO, DEW, eating frequency, and nutrient intake of shift workers across day types (including morning shifts, night shifts, and days off), and (2) investigate if chronotype further influences temporal eating patterns and diet composition across day types. We hypothesize that food intake on night shifts will be of poorer nutrient composition, with later times of food intake and greater eating frequency over a prolonged period due to a longer wake window, and that morning chronotypes will deviate from their early patterns of food intake when exposed to night shift work. Understanding changes in dietary habits of shift workers across different shift types and days off, and whether dietary behaviours adjust across these shifts based on chronotype will allow for more targeted health messaging to shift workers.

## 2. Materials and Methods

Data used in this study were obtained from baseline assessments of participants recruited for the Shifting Weight using Intermittent Fasting in night shift workers (SWIFt) study, a three-arm multisite 18-month parallel randomized controlled trial conducted at Monash University (Notting Hill, Melbourne) and the University of South Australia (City East Campus, Adelaide) [29]. Eligible participants were aged between 25 and 65 years, body mass index (BMI) was ≥28 kg/m^2^ for non-Asian men and women and ≥26 kg/m^2^ for Asian men and women, and worked a minimum of two nights per week; exclusion criteria, recruitment strategies, and study procedures were previously detailed [29]. Given that data used in this study were derived from a weight loss trial, participants in this study were limited to individuals with overweight and obesity. Two weeks prior to their baseline clinic visit, participants were asked to record a 14-day work diary, 14-day sleep diary, and 7-day food diary to capture habitual lifestyle habits. Data of participants recruited for the SWIFt study were excluded from this analysis based on the following exclusion criteria: did not fill in chronotype questionnaire; food/sleep/work diary not returned/completed; did not include or had limited reporting of times across all seven days of food diary recording; did not include meal/snack labels across all seven days of food diary recording; had mismatched time periods of recording between work, sleep, and food diaries.

### 2.1. Work Diary

In the work diary, participants recorded the shift type they were rostered to and start and end times of each of their shifts for 14 days. Using work diaries, diet and sleep data were coded to belong to one of the following day types: morning shift (MS), 1st night shift (1stNS), subsequent night shifts (SNS), 1st day off after night shifts (1stDONS), or other days off (DO). The 1st night shift was distinguished from subsequent night shifts as they tend to fall within a longer wake window with shift workers adjusting to night work. Similarly, the 1st day off after night shift was distinguished from other days off as they tend to fall within a shorter wake window with shift workers returning from night work in the morning. Data from several day types were excluded, including afternoon shifts, first day off after afternoon shifts, night shift on the 7th day (i.e., last day) of food diary recording, days with no times, and days when participants reported not attending work due to being unwell (i.e., sick days). These choices were made as there were minimal afternoon shifts in our dataset, representing only a small percentage (4.7%) of food diary days; wake times may have been later on the first day off after afternoon shifts compared to morning shifts, thereby influencing eating patterns; and the last night shift in the food diary was excluded as reporting discontinued from midnight.

### 2.2. Sleep Diary

In the sleep diary, participants recorded the time they went to bed and the estimated time they took to fall asleep, which was used to calculate their estimated time of sleep, as well as their final wake time from that sleep episode. For each day type, wake window was calculated, being the duration between offset (i.e., wake time) of the main sleep period prior to the shift/rest day and the onset of the subsequent main sleep period (i.e., sleep time), with naps that may have occurred within the period not subtracted.

### 2.3. Food Diary

The 7-day food diary was recorded using a paper record or kept electronically using the ‘Research Food Diary App’ (Xyris Software, Brisbane, QLD, Australia). Participants were instructed by a study dietitian to provide details including time of food and beverage intake, portion sizes, cooking methods, and brands. Food diary data were entered into Foodworks V.10 (Xyris Pty Ltd.) [30] using the Australia Diet and Recipes Analysis (AUSFOODS 2019) database by research dietitians. This included date of food diary, type of food intake (main meal or snack), food/beverage item (including brand/cooking method), and time of food/beverage intake. Foods that were not in the Foodworks database were substituted with an equivalent alternative from the database to preserve data recorded for that day. All food and beverages were entered into Foodworks, but an eating occasion was defined to have a minimum of 210 kJ and at least 15 min apart from another (i.e., food/beverage intake ≤ 15 min apart were combined as one eating occasion). If the participant provided a time range to indicate a long eating occasion (e.g., 6 p.m.–10 p.m. for a birthday dinner), the midpoint of the range was taken.

For each day type, temporal patterns of eating and nutrient intake data were collected within the day’s wake window. After excluding ineligible day types, outcomes were obtained for each of the remaining days. Times of first and last eating occasions were used to calculate the duration of eating window. Time of largest eating occasion was identified based on the eating occasion with the greatest energy content (kJ). Meal frequency and snack frequency were determined from total eating occasions based on counts of participant-identified meals (e.g., breakfast, lunch, dinner, and/or supper) and snacks (food and beverage intake consumed between meals), and total eating frequency based on the sum of meal and snack frequency. Diet composition data were determined for each day (including eating occasions < 210 kJ) and the following variables were exported into Excel: total energy (kJ), protein (% total energy), total fat (% total energy), saturated fat (% total energy), carbohydrate (% total energy), fibre (g), and alcohol (g). For all temporal patterns of eating and nutrient intake variables, data points that were ±3 standard deviations from the mean were double-checked for errors; days where dietary intake were deemed implausible by the study research dietitian (Y.Y.P.) were excluded from analysis.

### 2.4. Chronotype Questionnaire

At their baseline clinic visit, participants filled in the Composite Scale of Morningness (CSM), a circadian rhythm questionnaire that consists of 13 questions around an individual’s preference for timing of various activities, including timing of wake, sleep, and mental and physical tasks, as well as the degree of alertness and tiredness at different times of the day. Each item may be scored between either 1 and 4 or 1 and 5 depending on the item, with a higher number indicating a greater tendency for morningness. Individual items’ scores are summed to generate a total chronotype score to categorize individuals as either morning type (score > 44), intermediate type (score 23–43), or evening type (score ≤ 22). In this study, participants’ chronotype score was assessed on a continuous scale such that evening chronotypes had lower scores and morning chronotypes had higher scores [31].

### 2.5. Statistical Analysis

Demographic information including age and BMI was described using means and standard deviations while gender was described by counts and percentages. To examine differences in work and sleep between day types, linear mixed effects models were used; work start time, work end time, work duration, wake time, sleep time, and wake window were specified as dependent variables, and day type (MS, 1stNS, SNS, 1stDONS, DO) as a fixed effect, with a repeated effect of day, and a random effect of subjectID on the intercept.

Differences in temporal patterns of eating and diet composition were examined between day types using linear mixed-effects models (for continuous dependent variables) and generalized estimating equations (for counts and binary dependent variables). Dependent variables for linear mixed-effects models included FEO, LEO, DEW, LarEO, energy, fibre, and % energy from protein, fat, saturated fat, and carbohydrate. Dependent variables for generalized estimating equations included meal, snack, total frequency (using poisson loglinear), and alcohol (using binary logistic). Alcohol intake was dichotomized to the presence or absence of alcohol on each day type due to the large number of days with no alcohol consumption reported (626 days) and were presented as raw percentages rather than values from models. Day type (MS, 1stNS, SNS, 1stDONS, DO) was specified as a fixed effect, controlling for gender, age, BMI, and chronotype score (continuous), with a repeated effect of day, and a random effect of subjectID on the intercept. The Results section provides results of F-tests and estimated marginal means (EMM) evaluated for each shift type and at the mean (±1 SD) of chronotype score. Where significant, the day type*chronotype interaction was further investigated using tests of simple effects to examine the relationship between chronotype and the dependent variable at each level of day type and visualized using an interaction plot. Denominator degrees of freedom were corrected with the Satterthwaite approximation and are reported to the nearest whole number. In all analyses, results were considered statistically significant if *p* < 0.05.

## 3. Results

**Figure 1:**
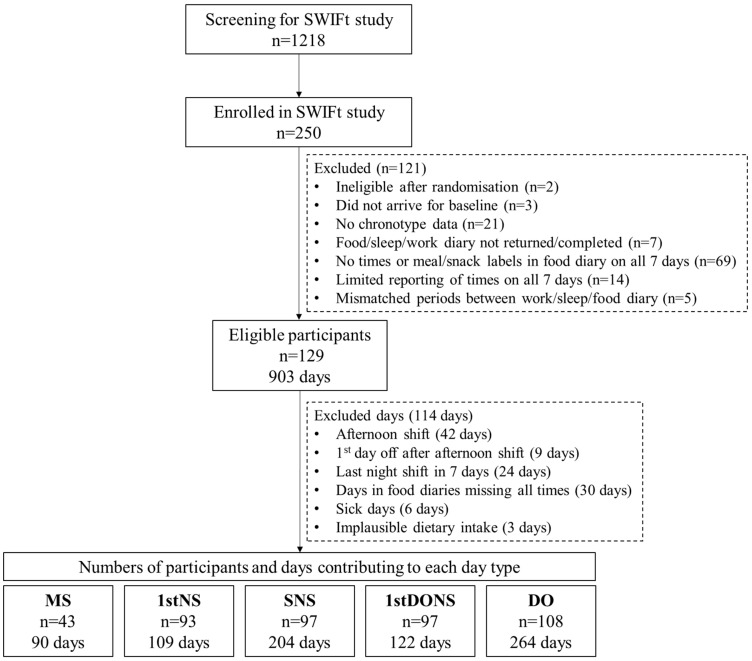
Flow chart of number of participants screened and enrolled in the SWIFt study, eligible for this study, and contributing participant numbers and days of sleep, work, and food diaries across each day type. MS: morning shift (n = 43 participants contributing 90 days of data); 1stNS: 1st night shift (n = 93 participants contributing 109 days of data); SNS: subsequent night shift (n = 97 participants contributing 204 days of data); 1stDONS: 1st day off after night shift (n = 97 participants contributing 122 days of data); DO: other days off (n = 108 participants contributing 264 days of data). Mean age and BMI of participants included in this analysis were 46.6 ± 10.2 years and 33.9 ± 5.6 kg/m^2^ (n = 129) and 56% identified as Female. The demographics of participants who contributed data for each shift/day type are shown in Table 1. Work duration on morning shifts were approximately 1.5 h shorter than on night shifts (*p* < 0.05). Wake windows were significantly different across each of the day types, with wake window around the 1st night shift being the longest (almost 24 h), and wake window on the first day off after night shifts the shortest (~11 h) (Table 1).

Figure 1 shows the participant flow chart of screening for and enrolment into the SWIFt study through to eligibility for this study. Of the participants enrolled in the SWIFt trial (n = 250), a proportion of food diaries were ineligible for inclusion in this study. This was due to many participants choosing to use the Research Food Diary App, which required timings to be typed in a separate note section rather than entered conveniently at the same time of food entry, leaving 129 individuals with eligible data specifically for this study. Strict definitions of eligible day types were applied, and completeness of meal timing data was required to ensure rigorous data analyses. There was no minimum cut-off of number of days of dietary data reported for inclusion in the analysis. Over 95% of participants provided dietary data for at least 4 days (Online Resource 1). In total, there were data for 90 MS, 109 1stNS, 204 SNS, 122 1stDONS, and 264 DO.

**Figure 2:**
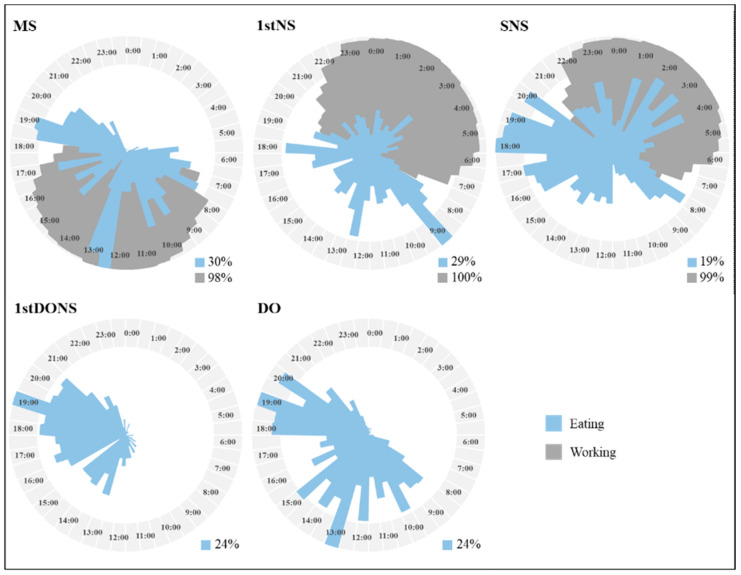
Rose charts showing the percentage of eating occasions (food or beverage of ≥210 kJ) or work occurring at each 30 min time point within the 24 h clock for each shift and day type. For each rose chart, the centre of the circle denotes no eating or working occasions reported (0 percentage (%)), and the proportion of participants reporting eating occasions (blue) and work (dark grey) is shown by the length of shading in 30 min intervals over 24 h. The most common eating or working occasions are identified by the longest line reaching the outermost circle. The percentage reported in the bottom-right next to each rose chart represent, the highest percentage of participants reporting they were eating or working during a 30 min interval. MS: morning shift; 1stNS: 1st night shift; SNS: subsequent night shift; 1stDONS: 1st day off after night shift; DO: other days off.

The percentages of individuals eating and working within each 30 min period over the 24 h clock for each day type are depicted in Figure 2. In morning shifts, food intake mostly occurred between 05:00 h and 21:00 h, with a peak number of eating occasions around 12:00 h–13:00 h, followed by 19:00 h, typical of lunch and dinner times. In night shifts, in contrast to morning shifts, food intake occurred around the 24 h clock. Patterns in timing of food intake differed between first night shifts and subsequent night shifts. In first night shifts, peaks in eating occasions occurred at 09:00 h, followed by 12:30 h and 18:00 h, with some eating occasions intermittently throughout the night. In subsequent night shifts, however, peaks in eating occasions occurred between 18:00 h and 20:00 h, followed by similarly large numbers of eating occasions at 17:00 h, 02:00 h, 03:00 h, 04:00 h, and 08:00 h. On first days off after night shift, food intake was spread out over a shorter window of time than other days off, with one peak in eating occasions at 19:00 h and similarly large numbers throughout the day between 13:00 h and 20:30 h. On other days off, however, peaks in eating occasion occurred at 13:00 h and 18:00 h–20:00 h, followed by similarly large peaks at 10:00 h, 12:00 h, 14:00 h, and 15:00 h.

**Table 1:** Demographic details of individuals contributing to each day type, and work and wake windows on each day type.

	MS	1stNS	SNS	1stDONS	DO
n	43	93	97	97	108
Female	14 (33%)	52 (56%)	56 (58%)	56 (58%)	61 (56%)
Age (years)	45.0 ± 9.8	47.2 ± 10.2	47.5 ± 10.0	47.4 ± 9.7	46.0 ± 10.3
BMI (kg/m^2^)	33.2 ± 4.0	33.7 ± 5.1	33.9 ± 5.4	33.8 ± 5.8	34.1 ± 5.9
Work					
Work start time (hh:mm)	08:29 ± 00:13 ^a^	19:55 ± 00:12 ^b^	20:00 ± 00:12 ^b^	-	-
Work end time (hh:mm)	17:38 ± 00:11 ^a^	06:49 ± 00:09 ^b^	06:58 ± 00:08 ^b^	-	-
Work duration (hh:mm)	09:21 ± 00:15 ^a^	10:52 ± 00:12 ^b^	10:57 ± 00:11 ^b^	-	-
Sleep					
Wake up time (hh:mm) *	06:00 ± 00:19 ^a^	08:51 ± 00:16 ^b^	13:57 ± 00:14 ^c^	12:56 ± 00:16 ^d^	08:04 ± 00:13 ^e^
Sleep onset (hh:mm) ^#^	23:26 ± 00:19 ^a^	08:00 ± 00:16 ^b^	08:53 ± 00:13 ^c^	23:48 ± 00:16 ^a^	23:35 ± 00:12 ^a^
Wake window duration (hh:mm) ^^^	17:21 ± 00:18 ^a^	23:14 ± 00:17 ^b^	18:49 ± 00:13 ^c^	10:56 ± 00:16 ^d^	15:46 ± 00:12 ^e^

MS: morning shift; 1stNS: 1st night shift; SNS: subsequent night shift; 1stDONS: 1st day off after night shift; DO: other days off; BMI: body mass index. Mean and standard deviation were reported except for gender, where n (%) was reported. * End of main sleep period preceding shift/rest days; ^#^ Estimated start of main sleep period following shift/rest days; ^^^ Duration of time between wake time and sleep time, including naps that are taken within the period. Estimated marginal mean (EMM) and standard error are reported for work and sleep variables. Results of post hoc analyses of pairwise comparisons of EMM between shifts where F values were significant; superscripts of different letters indicate significant differences (*p* < 0.05).

### 3.1. Day Type

Across day types, there were significant differences in times of FEO, LEO, LarEO, DEW, meal frequency, total eating frequency, total energy intake, % energy from carbohydrate, and fibre intake (*p* < 0.05) (Table 2). Table 3 shows temporal patterns of eating and nutrient intakes across shift/day types; where F/Wald χ^2^ values for day type were significant in Table 2, post hoc analyses identifying significant differences were reported. Time of FEO was earliest on MS at 07:37 h (*p* < 0.001) and latest on SNS and 1stDONS (*p* < 0.001), occurring in the afternoon at around 14:30 h. Time of LEO was not significantly different between MS and all days off, occurring between 20:00 and 21:00 h, and was significantly earlier by at least 6 hours than 1st and subsequent night shifts, where LEO occurred between 03:00 h and 05:00 h (*p* < 0.001). Eating window durations were generally longer on shift days than days off. The longest eating window occurred on 1stNS, spanning 16 h (*p* < 0.001), and shortest on 1stDONS at 6 h (*p* < 0.001), and were not significantly different between MS and SNS, being approximately 13 h. Time of LarEO occurred earliest at 16:13 h on MS (*p* < 0.05), not significantly different from DO, and latest on SNS at 20:09 h (*p* < 0.001). Meal (three eating occasions) and total eating frequency (five eating occasions) were not significantly different between MS and 1stNS but were significantly greater than other day types (*p* < 0.05), and lowest on 1stDONS (*p* < 0.001).

Energy intake was lowest on 1stDONS at around 7500 kJ (*p* ≤ 0.001) and greatest on DO, MS, and 1stNS at >9000 kJ, and not statistically different from each other. Energy intake was also not statistically different between MS and SNS (*p* = 0.393). There were no statistically significant differences in percentages of energy intake from protein, fat, and saturated fat across the day types. Fibre intake was greatest on 1stNS at 25g (*p* ≤ 0.001) and lowest on 1stDONS at 16 g (*p* ≤ 0.005), compared with ~20 g on MS, SNS, and DO.

### 3.2. Chronotype

By chronotype, there were significant differences in times of FEO, LEO, LarEO, and % energy from fat and saturated fat (*p* < 0.05) (Table 2). There was an inverse relationship between chronotype score with timing of FEO, LEO, LarEO, fat intake, and saturated fat intake, such that individuals who were a more extreme morning chronotype (higher chronotype score) had an earlier time of FEO, LEO, LarEO, and lower fat and saturated fat intake (Table 4).

### 3.3. Day Type by Chronotype

**Figure 3:**
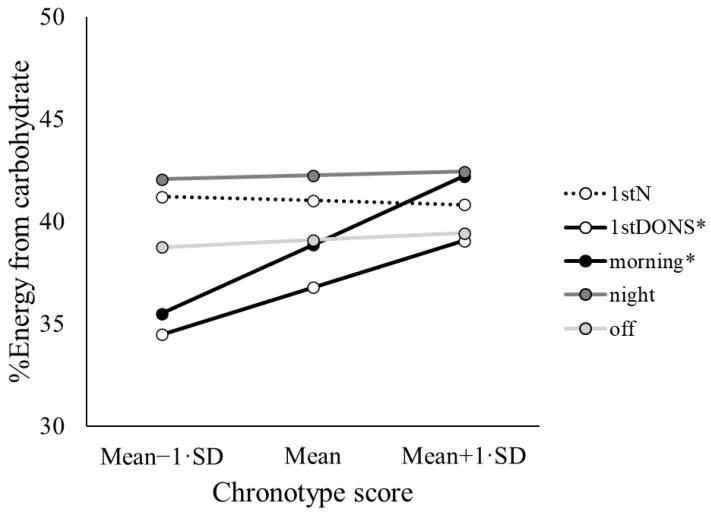
Shift type by chronotype interaction plot for %energy from carbohydrate. Values are estimated marginal means from the model evaluated at the mean value for chronotype plus or minus one standard deviation. MS: morning shift; SNS: subsequent night shift; 1stNS: 1st night shift; 1stDONS: 1st days off after night shift; DO: other days off. * *p* < 0.05 from simple effects test (slope is significantly different from zero).

There was a significant interaction between day type and chronotype for %energy from carbohydrate (*p* = 0.027) (Table 2). There was a significant, positive relationship between chronotype and %energy from carbohydrate for 1stDONS (*p* = 0.040) and MS (*p* = 0.004), where a higher chronotype score (greater morningness) was associated with greater %energy intake from carbohydrate (Figure 3).

## 4. Discussion

In our cohort of night shift workers, we found, as hypothesized, that temporal patterns of eating differed between shift types such that timing of first, last, and largest eating occasions were later on all night shifts than in the morning shift. However, duration of eating window was not different between morning shift and subsequent night shift. Additionally, meal frequency and total eating frequency were similar between first night shift and morning shift, and lower on subsequent night shifts. When differentiating first night shift from subsequent night shifts, and first day off after night shifts from days off, we observed significant differences in times of first and largest eating occasions, duration of eating window, eating frequency, and energy and fibre intake. Contrary to our hypothesis, dietary composition was not poorer on night shifts. There were no differences in total energy intake on day off, morning shift, and first night shift, but less energy was consumed on first day off after night shift. Across all day types, percentage of energy from fat and saturated fat, whilst similar, exceeded national guideline recommendations [32]. Percentages of energy from carbohydrate and total fibre intake were below recommended levels. In addition, whilst greater morningness predicted earlier timing of first, last, and largest eating occasions, and lower fat and saturated fat intake amongst shift workers, shift workers of different chronotypes did not have differing temporal patterns of eating or diet composition on each day type except for percentage of carbohydrate intake on morning shift and first day off after night shift.

### 4.1. Night-Time Energy Loading, Implications, and Considerations

From the second night shift onwards, no differences were observed in the duration of eating window or energy intake compared to morning shifts. This finding is in line with previous a systematic review that also reported no difference in energy intake of rotating shift workers on day and night shifts [33] and a study of Australian rotating shift workers by Shaw et al. from different industries [2]. Importantly, the key difference we observed on night shifts in comparison to other day types was the presence of eating occasions throughout the late night and early morning hours (approximately 23:00 h–05:00 h). This is in line with observations of shift workers in previous studies who were garbage collectors [3], police officers [4], or from mixed industries [2], with BMI ranging from the normal weight to overweight and obese categories. Additionally, in our study late night eating on night shifts did not translate to greater eating frequency compared to morning shifts or rest days, which has also been reported previously by others [2,4], highlighting redistribution of eating occasions to the night time in night shifts. This delayed pattern of food intake, coupled with time of largest eating occasion being the latest on subsequent night shift (~20:00 h), suggests later energy loading in night shifts.

As energy intake throughout the night is a distinguishing factor of night shifts, minimizing food intake through the night may improve cardiometabolic health markers given the circadian rhythm of energy metabolism [34]. A shift work study reported 12 weeks of time-restricted eating (food intake limited to 10 h during the day) led to improved HbA1c and diastolic blood pressure compared to baseline in firefighters with pre-existing cardiometabolic disease risk working 24 h shifts [35]. However, amongst night shift workers in a 4-week randomized controlled crossover trial, avoiding food intake between 01:00 h and 06:00 h led to small improvements in weight, but no differences in postprandial triglyceride and glucose response was detected compared to control [36]. Additionally, in another randomized crossover controlled trial, ten male police officers who fasted during two nights shifts (no food intake after 22:00 h) experienced lower insulin and Homeostatic Model Assessment for Insulin Resistance (HOMA-IR) the next morning and greater energy intake during an ad libitum test meal at 06:30 h compared to eating a meal (678 ± 42 kcal) at 02:00 h [37]. Beyond metabolic outcomes, other factors to consider in adjusting food intake during night shifts include shift workers using food as a strategy to maintain camaraderie and to stay alert at work [38]. Indeed, previous simulated shift work studies have demonstrated a late-night snack (00:30 h) improves performance and reduces sleepiness compared to consuming a meal or no food [39,40]. Hence, apart from minimizing nighttime food intake on night shifts to optimize cardiometabolic health, future dietary interventions should also investigate the feasibility and practicality of recommendations.

### 4.2. Nutrient Intake in the Context of Shift Type

Our findings revealed that across all day types, percentage of energy from fat and saturated fat exceeded the Acceptable Macronutrient Distribution Range (20–35% of energy for fat and >10% of energy for saturated fat), and percentage of energy from carbohydrate and fibre intake were below recommended levels [32]. The latest Australian National Nutrition Survey (NNS) in 2023 reported that the percentage of energy intake from fat was 32.4% and from carbohydrate 43.5% [41]. These data may have been influenced by the fact the participants in this study had BMIs in the overweight or obese category and may not be reflective of the diets of all Australian shift workers. Nonetheless, obesity is a common chronic condition in shift-working populations [42] and all shift workers may benefit from strategies that involve re-distribution of energy intake from fat and saturated fat towards carbohydrate, with a focus on choosing higher fibre-sources. Educating shift workers to increase fibre and protein intake at mealtimes and to consume low-energy, high-fibre snacks such as fruits and vegetables will allow for a more balanced macronutrient composition, while facilitating sustained energy release through night shifts to minimize nighttime food intake. Importantly, our study suggests that this advice is consistent regardless of day type.

A unique feature of this study was distinguishing the first night shift from the subsequent night shift, and the first day off after night shifts from days off within a wake window encompassing a shift. These categorisations provided insight into the influence of wake window on eating patterns to better target dietary recommendations. Our findings show that the transition to the first night shift involves a longer wake window of close to 24 h, resulting in a longer duration of eating window (16 h), and greater eating frequency and energy intake than subsequent night shifts. This is supported by an observational study of mining shift workers over an entire rotating shift cycle (mean BMI: 28.43  ±  3.73 kg/m^2^) which similarly found that within a wake window, transition to the first night shift had the longest eating window and the greatest energy intake compared to the other shifts [43]. These findings demonstrate that the longer wake window leading into the first night shift is a potential for greater energy consumption and reinforce the need to focus on dietary advice for this particular day type. Conversely, on the first day off after a night shift, wake time is much later (~13:00 h), with a shorter wake window, and associated shorter duration of eating window, lower eating frequency, energy and fibre intake, and percentage energy from carbohydrate compared to other days off. These highlight shift workers’ priority for recovery sleep and indicates that the optimization of nutrient intakes discussed in the previous paragraph may be more practical on the other day types.

### 4.3. Considering Shift Worker Chronotype

This study is one of a limited number of studies considering chronotype when investigating shift workers’ food consumption habits. In our cohort of shift workers, later chronotypes were associated with later times of the first eating occasion, which is typical of evening chronotypes, who tend to skip breakfast and have meals later [12,15], suggesting that when shift type is controlled for, temporal eating patterns by chronotypes are consistent between shift and non-shift workers. This is similar to a study of 123 rotating shift workers in Japan, which reported that a more pronounced evening chronotype was associated with a more irregular timing of eating based on a Japanese Eating Behaviour Questionnaire [27]. Interestingly, these authors also found no significant interactions between shift type and chronotype on temporal patterns of eating [27], which supports the finding in the present study. The fact that chronotype did not modulate the relationship between shift type and temporal aspects of eating suggests that morning and evening chronotypes do not behave differently when exposed to shift work and that shift type may be a greater predictor of timing of food intake than chronotype.

This finding was unexpected as chronotypes has been suggested to manifest differing phases in circadian rhythmicity. Stutz et al. previously demonstrated that chronotypes differ in diurnal glycemic response, where early chronotypes exhibited significantly greater 2 h postprandial glucose response to a high glycemic index meal at 8 p.m. as compared to 7 a.m., while no difference in glycemic response was exhibited between the two time points amongst evening chronotypes [44]. While these results were demonstrated only within group differences amongst morning chronotypes, they suggest that if morning chronotypes who perform shift work lose their inclination for earlier timing of food intake and exhibit late-night eating patterns like evening chronotypes, they could face greater risks to health. Mazri and colleagues’ study supports this point in an observational study of metabolically healthy versus metabolically unhealthy (higher adiposity, fasting blood glucose, insulin, triglycerides, and blood pressure) individuals with overweight and obesity [45]. The study reported morning chronotypes face an eight times greater risk of being metabolically unhealthy when consuming the lowest percentile of energy intake during an early window compared to consuming the highest percentile, and a four times greater risk of being metabolically unhealthy when consuming energy intake greater than the 75th percentile during a late window [45]. These increased risks were not observed by evening chronotypes in the study. Hence, there is a need for future shift work studies to confirm if both morning and evening chronotypes display similar late-night food intake patterns exposed to night shift work, and if so, whether metabolic response to night-time food intake are similar between them, which will increase the cardiometabolic risk of morning chronotypes engaged in shift work.

### 4.4. Strengths and Limitations

This study has several limitations. Firstly, the study population consists of individuals with overweight and obesity, hence study findings may not be extrapolated to the general shift work population, but it serves to provide insight into the behaviours of shift workers with elevated adiposity. Secondly, the cross-sectional nature of our study design prevents causation to be determined; our findings require confirmation in future shift-work studies by tracking dietary patterns over a more prolonged period using convenient time-stamped phone applications while exploring behavioural motivators. Thirdly, the lack of prompting by the ‘Research Food Diary App’ for the timing of eating occasions reduced the size of dataset available for analysis. Additionally, while the Composite Scale of Morningness was convenient, it has not been validated amongst shift workers; the Munich Chronotype Questionnaire for shift workers, which accounts for shift types in identifying chronotype, may be more relevant for this population [46].

However, there are several strengths of this study to note. Firstly, a unique strength was separately studying differences in dietary patterns between first and subsequent night shifts, and days off that immediately follow a night shift with other days off, which allowed distillation of food consumption patterns once the pattern of night shift work was established and, on a day off uninfluenced by a previous night shift, a factor not commonly distinguished in other shift work studies. Secondly, our definition of an eating occasion based on a minimum energy criterion with a 15 min time interval was previously described to best predict variance in energy intake amongst Australian adults [47]. Finally, this study is one of few studies that has focused on characterizing the temporal patterns of eating while considering the influence of chronotype in a diverse group of free-living shift workers.

## 5. Conclusions

Our study of night shift workers in Australia with overweight and obesity demonstrated that energy intakes were similar across morning shifts, first night shifts, and other days off when based on the wake window. Also similar were total eating frequency between morning shifts and first night shifts and duration of eating window between morning shifts and subsequent night shifts. The main difference between night shifts and morning shifts/days off was a greater distribution of food intake towards night-time hours, highlighting the need for future studies to investigate the metabolic benefits and practicality of redistributing energy intake towards earlier parts of the night minimizing energy intake during night shift work. Regardless of shift or day type, a redistribution of energy intake from fat and saturated fat towards carbohydrate, and an increase in fibre would improve the diet of night shift workers. There was little interaction between chronotype and shift type on temporal eating patterns and dietary composition. Further studies are needed to confirm if the influence of shift work overrides that of chronotype in dictating the dietary habits of shift workers, and if so, whether a mismatch between shift type and chronotype (e.g., morning chronotypes working night shifts) places an individual at even greater cardiometabolic health risks.

## Figures and Tables

**Table 2:** Results from Linear Mixed Models/Generalized Estimating Equations analysis showing the main effects of day type and chronotype, and the interaction effects of day type by chronotype.

	Day Type		Chronotype		Day Type × Chronotype	
	F/Wald χ^2^_(df)_	*p*	F/Wald χ^2^_(df)_	*p*	F/Wald χ^2^_(df)_	*p*
**Temporal patterns of eating**						
FEO	11.03_(4,730)_	**<0.001**	13.22_(1,130)_	**<0.001**	1.94_(4,729)_	0.102
LEO	49.03_(4,686)_	**<0.001**	6.86_(1,123)_	**0.010**	0.96_(4,687)_	0.428
DEW	10.41_(4,700)_	**<0.001**	0.03_(1,134)_	0.863	1.54_(4,696)_	0.188
LarEO	2.80_(4,708)_	**0.025**	3.99_(1,129)_	**0.048**	0.692_(4,703)_	0.597
Meal frequency ^#^	29.54_(4)_	**<0.001**	3.17_(1)_	0.075	7.30_(4)_	0.121
Snack frequency ^#^	8.65_(4)_	0.070	2.17_(1)_	0.141	5.06_(4)_	0.281
Total eating frequency ^#^	15.94_(4)_	**0.003**	0.33_(1)_	0.568	7.02_(4)_	0.135
**Diet composition**						
Energy (kJ)	2.88_(4,726)_	**0.022**	0.12_(1,128)_	0.728	1.13_(4,727)_	0.342
Protein (%E)	2.19_(4,732)_	0.069	1.28_(1,132)_	0.259	2.09_(4,731)_	0.081
Fat (%E)	1.63_(4,733)_	0.164	7.18_(1,134)_	**0.008**	1.19_(4,732)_	0.313
Sat fat (%E)	0.5_(4,727)_	0.736	5.91_(1,132)_	**0.016**	0.46_(4,727)_	0.762
Carbohydrate (%E)	4.16_(4,730)_	**0.002**	3.42_(1,134)_	0.067	2.76_(4,731)_	**0.027**
Fibre (g)	4.75_(4,724)_	**<0.001**	0.19_(1,128)_	0.666	2.29_(4,725)_	0.058
Alcohol (yes/no)	1.71_(4)_	0.790	0.05_(1)_	0.832	1.64_(4)_	0.802

FEO: first eating occasion; LEO: last eating occasion; DEW: duration of eating window; LarEO: largest eating occasion; %E: percentage of total energy intake. Linear mixed effects models were used except for variables marked ^#^, where generalized estimating equations were used; gender, age, and BMI were controlled for in all models.

**Table 3:** Temporal patterns of eating and diet composition across day types.

	MS ^a^		1stNS ^b^		SNS ^c^		1stDONS ^d^		DO ^e^	
	EMM	SE	EMM	SE	EMM	SE	EMM	SE	EMM	SE
**Temporal patterns of eating**										
FEO (hh:mm)	7:37 ^a^	0:22	10:32 ^be^	00:18	14:33 ^cd^	00:14	14:23 ^cd^	00:17	09:57 ^be^	00:13
LEO (hh:mm)	20:40 ^ade^	00:27	03:21 ^b^	00:21	04:54 ^c^	00:18	20:34 ^ade^	00:21	20:09 ^ade^	00:17
DEW (hh:mm)	12:56 ^ac^	00:31	16:15 ^b^	00:25	13:42 ^ac^	00:20	06:08 ^d^	00:24	10:24 ^e^	00:18
LarEO (hh:mm)	16:13 ^ae^	00:33	17:42 ^bd^	00:27	20:09 ^c^	00:20	18:05 ^bd^	00:26	16:35 ^ae^	00:18
Meal frequency	2.8 ^ab^	0.1	2.9 ^ab^	0.1	2.2 ^c^	0.1	1.7 ^d^	0.1	2.6 ^e^	0.0
Snack frequency	2.4	0.3	2.1	0.2	2.2	0.1	1.5	0.1	1.9	0.1
Total eating frequency	5.2 ^ab^	0.3	5.1 ^ab^	0.2	4.4 ^ce^	0.1	3.2 ^d^	0.2	4.4 ^ce^	0.1
**Diet composition**										
Energy (kJ)	9152.7 ^abe,ac^	446.1	9596.6 ^abe^	362.2	8744.0 ^ac^	308.6	7492.3 ^d^	351.7	9599.2 ^abe^	287.1
Protein (%E)	20.7	0.8	20.3	0.6	20.2	0.5	18.9	0.6	18.5	0.5
Fat (%E)	35.5	1.1	35.5	0.9	34.5	0.7	37.7	0.9	36.0	0.7
Sat fat (%E)	14.4	0.6	13.8	0.5	13.4	0.4	14.1	0.5	14.1	0.4
Carbohydrate (%E)	38.8 ^ab,ad,ae^	1.3	41.0 ^ab,bc,be^	1.0	42.2 ^bc^	0.9	36.8 ^ad^	1.0	39.1 ^ae,be^	0.8
Fibre (g)	20.2 ^ace^	1.3	24.9 ^b^	1.1	20.7 ^ace^	0.9	16.2 ^d^	1.0	21.2 ^ace^	0.8
Alcohol (% yes) ^^^	20%		8.3%		6.9%		27.9%		33.3%	

MS: morning shift; 1stNS: 1st night shift; SNS: subsequent night shift; 1stDONS: 1st days off after night shift; DO: other days off. EMM: estimated marginal means; SE: standard error. FEO: first eating occasion; LEO: last eating occasion; DEW: duration of eating window; LarEO: largest eating occasion. %E: percentage of total energy intake. ^a, b, c, d, e^ Results of post hoc analyses of pairwise comparisons of EMM between shifts where F/Wald χ^2^ values were significant in Table 2; values with different superscripts are significantly different from each other (*p* < 0.05). ^^^ Percentage of days out of total days for each shift where alcohol was consumed, presented as raw percentages rather than values from models.

**Table 4:** Temporal patterns of eating and diet composition for mean ± 1 SD chronotype score.

	Mean − 1·SD		Mean		Mean + 1·SD	
	EMM	SE	EMM	SE	EMM	SE
**Temporal patterns of eating**						
FEO (hh:mm)	12:04	00:15	11:24	00:10	10:45	00:14
LEO (hh:mm)	14:33	00:20	13:55	00:14	13:17	00:19
DEW (hh:mm)	11:51	00:19	11:53	00:13	11:56	00:18
LarEO (hh:mm)	18:11	00:19	17:45	00:13	17:18	00:18
Meal frequency	2.3	0.1	2.4	0.0	2.5	0.0
Snack frequency	2.2	0.1	2.0	0.1	1.9	0.1
Total eating frequency	4.5	0.2	4.4	0.1	4.4	0.2
**Diet composition**						
Energy (kJ)	9002.6	348.8	8917.0	237.1	8831.3	333.8
Protein (%E)	19.3	0.6	19.7	0.4	20.2	0.5
Fat (%E)	37.3	0.8	35.8	0.5	34.4	0.7
Sat fat (%E)	14.8	0.5	14.0	0.3	13.1	0.5
Carbohydrate (%E)	38.4	0.9	39.6	0.6	40.8	0.9
Fibre (g)	20.3	1.0	20.6	0.7	20.9	1.0

EMM: estimated marginal means; SE: standard error. FEO: first eating occasion; LEO: last eating occasion; DEW: duration of eating window; LarEO: largest eating occasion. %E: percentage of total energy intake.

## Data Availability

The data that support the findings of this study are available from the corresponding author, Y.Y.P., upon reasonable request.
